# Protein Tyrosine Phosphatases as Potential Regulators of STAT3 Signaling

**DOI:** 10.3390/ijms19092708

**Published:** 2018-09-11

**Authors:** Mihwa Kim, Liza D. Morales, Ik-Soon Jang, Yong-Yeon Cho, Dae Joon Kim

**Affiliations:** 1 Department of Biomedical Sciences, School of Medicine, University of Texas Rio Grande Valley, Edinburg, TX 78539, USA; mihwa.kim@utrgv.edu; 2 Department of Human Genetics, School of Medicine, University of Texas Rio Grande Valley, Edinburg, TX 78539, USA; liza.moralessmith@utrgv.edu; 3 South Texas Diabetes and Obesity Institute, School of Medicine, University of Texas Rio Grande Valley, Edinburg, TX 78539, USA; 4 Division of Bioconvergence Analysis, Korea Basic Science Institute, Daejeon 305-333, Korea; jangiksn@kbsi.re.kr; 5 Integrated Research Institute of Pharmaceutical Science & BK21 PLUS Team for Creative Leader Program for Pharmacomics-based Future Pharmacy, College of Pharmacy, The Catholic University of Korea, Bucheon-si, Gyeonggi-do 420-743, Korea; yongyeon@catholic.ac.kr

**Keywords:** STAT3, PTPRT, PTPRD, SHP1, SHP2, TC-PTP

## Abstract

The signal transducer and activator of transcription 3 (STAT3) protein is a major transcription factor involved in many cellular processes, such as cell growth and proliferation, differentiation, migration, and cell death or cell apoptosis. It is activated in response to a variety of extracellular stimuli including cytokines and growth factors. The aberrant activation of STAT3 contributes to several human diseases, particularly cancer. Consequently, STAT3-mediated signaling continues to be extensively studied in order to identify potential targets for the development of new and more effective clinical therapeutics. STAT3 activation can be regulated, either positively or negatively, by different posttranslational mechanisms including serine or tyrosine phosphorylation/dephosphorylation, acetylation, or demethylation. One of the major mechanisms that negatively regulates STAT3 activation is dephosphorylation of the tyrosine residue essential for its activation by protein tyrosine phosphatases (PTPs). There are seven PTPs that have been shown to dephosphorylate STAT3 and, thereby, regulate STAT3 signaling: PTP receptor-type D (PTPRD), PTP receptor-type T (PTPRT), PTP receptor-type K (PTPRK), Src homology region 2 (SH-2) domain-containing phosphatase 1(SHP1), SH-2 domain-containing phosphatase 2 (SHP2), MEG2/PTP non-receptor type 9 (PTPN9), and T-cell PTP (TC-PTP)/PTP non-receptor type 2 (PTPN2). These regulators have great potential as targets for the development of more effective therapies against human disease, including cancer.

## 1. Introduction

The signal transducer and activator of transcription (STAT) protein family includes intracellular latent transcription factors that facilitate the cellular signaling initiated by stimuli from growth factors and cytokines that is required for multiple key cellular processes. Currently, there are seven known members of the STAT family in humans: STAT1, STAT2, STAT3, STAT4, STAT5a, STAT5b, and STAT6. Signal transducer and activator of transcription 3 (STAT3, encoded by the *STAT3* gene) is the most prominent of all the STATs, because it has been established as a major oncogenic protein. In a majority of cancers, it is constitutively activated at tyrosine 705 (Y705), which disrupts mechanisms involved in cell proliferation, autophagy, differentiation, and/or cell survival, depending on the cell type [1,2,3].

**Figure 1:**
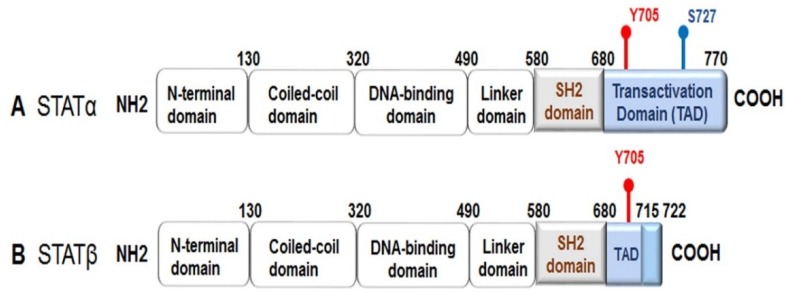
Schematic of the full-length signal transducer and activator of transcription 3 (STAT3) protein structure. STAT3 is comprised of six conserved domains: an N-terminal domain, a coiled-coil domain, a DNA-binding domain, a linker domain, a Src homology region 2 (SH-2) domain, and a C-terminal transactivation domain. The transactivation domain contains two phosphorylation (P) sites: a tyrosine residue (Y) at 705, which is required for STAT3 activation, and a serine residue (S) at 727. Once STAT3 is activated, the P(Y)–SH-2 domains interact to form a STAT3 dimer. (**A**) STAT3α, the full-length form of STAT3; (**B**) STAT3β is a C-terminal truncated form of STAT3 generated by an alternative splicing. STAT3β does not contain a serine phosphorylation site at 727.

All the STATs are composed of six conserved domains: an N-terminal domain, a coiled-coil domain, a DNA-binding domain, a linker domain, a Src homology region 2 (SH-2) domain, and a C-terminal transactivation domain. Alternative splicing produces two active isoforms of STAT3: the full-length form, referred to as STAT3α, and a truncated form named STAT3β, which lacks approximately 55 bases pairs within the C-terminal domain (Figure 1) [4,5]. STATs are activated by phosphorylation at a tyrosine residue (Y) and at a serine residue (S) within the transactivation domain. Once activated, a STAT makes a reversible binding with specific tyrosine residues on active cytokine or growth factor receptors through its SH-2 domain, which permits the Janus-activated kinases (JAKs) to facilitate tyrosine phosphorylation of the STAT. Phosphorylation triggers STAT dimerization by reciprocal phosphotyrosine–SH-2 interactions to form stable “parallel” dimers. The active STAT dimers can then be translocated into the nucleus where they bind to DNA elements within target genes to regulate transcriptional expression. All the STATs, except for STAT2, recognize consensus palindromic sequences (TTCN*_x_*GAA, where *x* = 2–6 nucleotides), known as γ interferon activation sites (GAS), within the gene promoter, and these sites determine the specificity of the STAT that binds to the promoter; for example, if the GAS has an *x* of N equal to 4, then STAT3 can bind more efficiently [3,6,7,8,9].

As previously mentioned, STAT3 is activated following phosphorylation at Y705 (Figure 1). STAT3 activation also can occur via phosphorylation of S727 by mitogen-activated protein kinases (MAPKs) or c-Src non-receptor tyrosine kinase [10,11]. Serine phosphorylation of STAT3 promotes transactivation [5,12,13]. Furthermore, it has been demonstrated that phosphorylated STAT3 (S727) can be transported into the mitochondria instead of the nucleus, where it contributes to cellular homeostasis by modulating the optimal function of the electron transport chain [14,15,16,17]. Interestingly, STAT3β lacks S727, and due to this truncation, it has been regarded as a dominant negative form; however, evidence from functional in vivo studies has revealed that STAT3β can not only activate STAT3 target genes, but it also plays a role in inflammation [5].

The rate and duration of phosphotyrosine-based signaling, such as the JAK-mediated activation of STAT3, is maintained by the activities of protein tyrosine phosphatases (PTPs). PTPs are a large and diverse family of enzymes, which catalyze the removal of the phosphate group from the tyrosine residue of phosphorylated proteins [18,19,20,21]. Despite the importance of PTPs in the regulation of STAT signaling, particularly in the development of a variety of human diseases, such as cancer, the relationship between PTPs and STATs has still not been completely characterized. Here, we will review the PTPs that have been implicated in the inactivation of STAT3, with an emphasis on PTP regulation of STAT3 signaling in cancer.

## 2. Protein Tyrosine Phosphatase Receptor-Type D

PTP receptor-type D or PTPRD (PTPδ) is a member of the highly conserved family of receptor PTPs that is encoded by the *PTPRD* gene. It is classified as a member of the LAR subfamily of transmembrane PTPs, which includes LAR and PTPσ (PTPRS) [22,23]. PTPRD is a transmembrane protein composed of an extracellular region, which possesses three Ig-like and eight fibronectin-type III-like domains, a single transmembrane region, and two tandem intracytoplasmic catalytic domains, referred to as D1 and D2. The extracellular region is similar in structure to cell adhesion molecules, implying that PTPRD, and other receptor-type PTPs like it, have similar ligands and functions [23,24]. In fact, PTPRD is known to be a ligand for cell adhesion and neurite outgrowth, and different truncated isoforms can be produced through alternative splicing, depending on the requirements of the different tissue and developmental stages [25]. PTPRD is expressed in astrocytes and tissue, such as brain, colon, and breast tissues [26].

Receptor-type PTPs have been shown to be inactivated in a number of human cancers, implying that these PTPs have tumor suppressive capabilities. The *PTPRD* gene, specifically, is frequently inactivated by genetic (deletion, mutation, copy number loss) or epigenetic (hypermethylation) mechanisms in cancers, such as glioblastoma multiforme (GBM), colon cancer, breast cancer, neuroblastoma, lung cancer, and squamous cell carcinoma (SCC) [24,26,27,28,29,30,31,32]. These findings indicate that PTPRD is a major tumor suppressor, and in fact, functional studies have revealed that PTPRD-deficiency enhanced the tumor-forming capability of immortalized human astrocytes in mouse xenograft models [26]. Another study showed that homozygous or heterozygous deletion of *Ptprd* in the absence of *Cdkn2a*, a gene that encodes the tumor suppressors p16^Ink4^ and p14/p19^Arf^, resulted in increased tumorigenesis in comparison to mice with *Cdkn2a* deletion alone, and *Ptprd*-deficiency may influence which types of tumors form [30]. PTPRD cooperates with CD44 and β-catenin/TCF signaling to regulate cell migration and progression in colon cancer [24]. CD44 is a major cell-surface glycoprotein, critical to cancer invasion and metastasis, which can function as a marker for cancer stem cells, cells that can give rise to the many cell types that form tumors. Additionally, it has been shown that the exogenous expression of PTPRD in PTPRD-deficient primary melanoma cells significantly decreased cell growth and cell viability, and it yielded an increase in apoptosis in a time-dependent manner; however, the expression of PTPRD containing cancer-specific mutations reversed this effect [29].

PTPRD is capable of directly interacting with PTPRS [22], the cytoskeletal remodeling proteins liprin-α-1 and MIM (Missing in Metastasis) [23,24], and STAT3 [26,30,33], which suggests that PTPRD may play a functional role in cancer cell survival, adhesion, and/or migration through the signaling mechanisms mediated by these substrates. Veeriah et al. demonstrated that PTPRD acts as a tumor suppressor through its ability to negatively regulate STAT3-mediated signaling [26]. The exogenous expression of PTPRD in human GBM and other cancer cell lines inhibited cell growth, which coincided with a substantial decrease in the expression levels of phosphorylated STAT3 (Y705) and, consequently, one of its downstream targets, suppressor of cytokine signaling 3 (SOCS3) [26]. However, the expression of PTPRD containing cancer-specific mutations reversed the inhibitory effect on cell growth and STAT3 phosphorylation. Immunoprecipitation assays confirmed that PTPRD directly interacts with STAT3 [26]. Heterologous loss of *Ptprd* or *PTPRD* is sufficient to see a significant increase in STAT3 phosphorylation and an upregulation of STAT3 target genes within GBM tumors [30]. Loss-of-function mutations in *PTPRD* contribute to enhanced cell growth and increased levels of phosphorylated STAT3 (Y705) in head and neck SCC cells [31]. Interestingly, head and neck SCC cells containing a *PTPRD* mutation were more susceptible to a STAT3 inhibitor, suggesting that use of this type of anti-cancer drug could be utilized for more successful treatments of head and neck SCC patients [31].

## 3. Protein Tyrosine Phosphatase Receptor-Type T

PTP receptor-type T (PTPRT), alternatively known as PTPρ, is another receptor PTP that belongs to the type IIB receptor-type (R2A) PTP subfamily, which also includes PTPRK (PTPκ), PTPRM (PTPµ), and PTPRU (otherwise known as PCP-2). It is composed of an extracellular domain containing a MAM (meprin/A5/PTPµ) domain and an Ig domain and four fibronectin-type III repeats, a transmembrane domain, a juxtamembrane region, and two phosphatase domains, referred to as D1 and D2. Primarily, D1 is responsible for the catalytic activity of PTPRT, whereas D2 is more important for regulation [34]. PTPRT is largely expressed in the brain and spinal cord. It plays a role in cell adhesion through its extracellular domain [35,36,37], and it serves an important function in neurological development. For instance, PTPRT has been shown to directly dephosphorylate E-cadherin at intercellular adheren junctions to regulate hemophilic cell–cell adhesion in the central nervous system [38]. Also, it stimulates synapse formation through its interaction with neuroligin and neurexin, proteins that connect and maintain the synapse between neurons [39].

Like PTPRD, PTPRT is inactivated by mutation in many cancers, including lung cancer, gastric cancer, and head and neck SCC, and it is most frequently mutated in colorectal cancer (CRC) [40,41,42,43]. Mutations in *PTPRT* consist of nonsense, insertion, and deletion mutations, with the majority being missense mutations. Missense mutations in the catalytic domain have been implicated in reducing its phosphatase activity, and mutations in the extracellular domain impair its function in cell adhesion [35,42,44]. PTPRT can function as a tumor suppressor through its interaction with its substrates, paxillin and STAT3 [40,41]. The knockout of PTPRT has resulted in increased colon tumor formation in a mouse model, which correlates with increased paxillin phosphorylation [44]. The activation of paxillin triggers the activation of AKT (protein kinase B), a major kinase involved in oncogenic signaling. Zhang et al. [41] demonstrated that PTPRT negatively regulates STAT3-mediated signaling by directly dephosphorylating STAT3 at Y705, which prohibits STAT3 nuclear translocation, resulting in a variety of effects depending on the cancer type. Whole exome sequencing and reverse-phase protein array analysis showed that *PTPRT* mutations are associated with increased STAT3 activation in head and neck SCC, leading to increased cell survival and, therefore, may serve as a biomarker to predict the efficacy of STAT3 inhibitors as a chemotherapeutic [43]. Many human cancers show aberrant hypermethylation of the *PTPRT* promoter, which results in the decreased expression of *PTPRT* mRNA [45]. Peyser et al. [45] showed that this decrease correlated with an increase in phosphorylated STAT3 in head and neck SCC and sensitivity to STAT3 inhibition. These findings suggest that *PTPRT* hypermethylation could also function as a biomarker for the efficacy of STAT3 inhibitors against cancer. Furthermore, it has been shown that post-translational modification of PTPRT by glycosylation reduces its activity by causing it to dimerize, which has been shown to result in enhanced cancer cell migration via the activation of STAT3-mediated signaling [46].

## 4. Protein Tyrosine Phosphatase Receptor-Type K

As previously mentioned, PTPRK (encoded by *PTPRK*) is a R2A subfamily member with PTPRT, and it functions in homophilic binding via its extracellular domain [47]. PTPRK is expressed in the cytoplasm of different tissues and cell types, including the central nervous system and human keratinocytes [48]. It has been shown to mediate neurite outgrowth via a Grb2/MEK1-dependent signaling pathway and T-cell development [49,50]. In keratinocytes, transforming growth factor β1 (TGF-β1) inhibits cell proliferation and triggers cell migration through the upregulation of PTPRK [48]. Additionally, PTPRK was found to co-localize with β-catenin at adherens junctions, suggesting that PTPRK plays a role in cell contact and adhesion, such as PTPRD and PTPRT [51,52]. Indeed, further studies have demonstrated, for example, that PTPRK negatively regulated adhesion and invasion in breast cancer cells [53]. The expression of *PTPRK* mRNA transcript was reduced in the primary tumors harvested from breast cancer patients with metastatic tumors or who had succumbed to the disease. Furthermore, patients who expressed higher levels of *PTPRK* survived longer than patients with low levels. In vitro studies confirmed that the knockdown of PTPRK increased cell proliferation, adhesion, and invasion, suggesting it functions as a tumor suppressor in breast cancer [53]. PTPRK has also been shown to act as a tumor suppressor in central nervous system lymphomas, CRC, and prostate cancer [54,55,56]. The use of mapping arrays revealed that genetic alterations (gene deletion and missense mutations) of *PTPRK* could be found in glioma biopsies, and they disrupt PTPRK activity and post-translational regulation [57].

Some major substrates of PTPRK that have been identified are epidermal growth factor receptor (EGFR) and STAT3. PTPRK can directly dephosphorylate EGFR during both basal- and ligand-stimulated EGFR phosphorylation, and it can contribute to tumor suppression through its negative regulation of EGFR signaling [58,59,60]. Chen et al. were the first to demonstrate that PTPRK directly interacts with and dephosphorylates active STAT3 at Y705 [61]. Their studies revealed that the expression of PTPRK was decreased in nasal-type natural killer T-cell lymphoma (NKTCL) human cell lines and primary tumors, and this decrease was inversely correlated with phosphorylated STAT3 (Y705) expression; the knockdown of PTPRK further decreased the expression levels of phosphorylated STAT3, whereas the overexpression of PTPRK reversed this effect, which resulted in increased NKTCL cell growth and invasion [61]. Additionally, Chen et al. [61] found that decreased PTPRK expression was a result of decreased PTPRK mRNA levels due to monoallelic deletion and promoter hypermethylation, and these genetic alterations were associated with a poor prognosis for NKTCL patients undergoing standard treatment.

## 5. Src Homology Region 2 Domain-Containing Phosphatase 1

Src homology region 2 (SH-2) domain-containing phosphatase 1, or SHP1, is a non-receptor PTP encoded by *PTPN6*. SHP1 is a member of a subfamily of non-transmembrane PTPs that are composed of two tandem N-terminal Src homolog domains (N-SH2 and D-SH2), a classic catalytic PTP domain, and a C-terminal tail containing two sites for tyrosine phosphorylation and a nuclear localization signal [62,63,64]. There are two forms of SHP1, which differ by their N-terminal amino acid sequences: form I contains MLSRG, and form II contains MVR. The expression of these isoforms are regulated by either promoter 1, which is found in nonhematopoietic-derived cells, or promoter 2, which is exclusively active in hematopoietic-derived cells [65]. SHP1 is most abundantly expressed in the nucleus of epithelial cells and the cytoplasm of hematopoietic cells, although stimulation by cytokines can induce nuclear translocation of SHP1 in hemopoietic cells [63]. The binding of SHP1 at its N-terminal SH-2 domain to a specific motif—the immune-receptor tyrosine-based inhibitory motif (IVLxYxxIVL)—in receptors, scaffold adapters, or immune inhibitory receptors causes a conformational change that actives SHP1 by exposing its active site to its substrates [63,66]. The modification of the C-terminal tail by truncation, phospholipid binding, or tyrosine phosphorylation has been implicated in the regulation of SHP1 phosphatase activity, and moreover, it was shown that Src kinase is one kinase that phosphorylates SHP1 in order to allow SHP1 to more readily dephosphorylate Src-activated substrates [67]. SHP1 is a major negative regulator of cytokine-mediated signaling pathways, such as inflammatory protein interleukin (IL)-3 receptor signaling and epidermal growth factor (EGF) signaling, in lymphocytes, which facilitate lymphocyte activation for cell proliferation and differentiation [66,68]. On the other hand, SHP1 has been implicated in the positive regulation of glia cell differentiation and Ras-dependent MAPK activation [67].

The downregulation or loss of SHP1 protein expression is a characteristic of human lymphoma and leukemia, and in fact, methylation of the *PTPN6* promoter, resulting in suppressed SHP1 expression, was found in anaplastic large cell lymphoma (ALCL), multiple myeloma, T-cell lymphoma, and B-cell lymphoma [68,69,70,71,72,73,74]. STAT3 and DNA methyltransferase 1 appear to function cooperatively to promote this epigenetic silencing in T-cell lymphoma [74]. Additionally, a recent study identified loss-of-function point mutations within *PTPN6* in diffuse large B-cell lymphoma [75]. Altered SHP1 expression has also been noted in breast, ovarian, prostate, and pancreatic cancers [65]. The tumor suppressive capability of SHP1 occurs through its regulation of JAK and STAT. For example, Han et al. [68] demonstrated that overexpression of SHP1 in ALK+ ALCL cells—a distinct type of non-Hodgkin lymphoma—which are deficient in SHP1, reversed JAK3 and STAT3 activation, which corresponded with a decrease in STAT3 targets. It was also shown that SHP1 downregulates JAK3 via increased proteasome degradation [68]. Research into the chemotherapeutic properties of various compounds have revealed the importance of SHP1 regulation of JAK/STAT signaling in the efficacy of these compounds. Guggulsterone (GS), a phytosteroid extracted from the guggul plant, can induce apoptosis and suppress the proliferation of multiple cancer types, such as leukemia, head and neck SCC, and melanoma. Ahn et al. demonstrated that GS induces SHP1 expression, which resulted in the inhibition of JAK2 activation and markedly decreased STAT3 phosphorylation [76]. Dovitinib, a multi-targeted receptor kinase inhibitor, was shown to directly and effectively induce SHP1 activity, leading to decreased STAT3 phosphorylation, the inhibition of hepatocellular carcinoma (HCC) growth, and an increase in apoptosis [77]. These findings further implied that the use of dovitinib in combination with the current clinical drug, sorafenib, could help overcome chemoresistance in HCC. However, a recent study was able to generate sorafenib derivatives that more effectively activate SHP1 activity and, thereby, inhibit STAT3 activation and suppress cancer cell growth to combat tumorigenesis and chemoresistance [78]. Plumbagin, a vitamin K3 analogue derived from a medicinal plant, induces the expression of SHP1 in human multiple myeloma cells, which resulted in the inhibition of STAT3 phosphorylation via the inactivation of c-Src, JAK1, and JAK2 [79]. In epidermal keratinocytes, SHP1 is initially activated in response to ultraviolet B (UVB) exposure and contributes to reduced STAT3 phosphorylation in cooperation with other PTPs, Src homology region 2 domain-containing phosphatase-2 (SHP2) and T-cell PTP (TC-PTP), implying that SHP1 is involved in an initial protective mechanism against UVB-induced skin cancer formation [80].

It is important to note that the modulation of SHP1 expression can play a role in noncancerous disease as well. Ruchusatsawat et al. [81] revealed that the *PTPN6* promoter 2 is methylated in normal epithelial cells and tissues to repress SHP1 expression; however, a significant increase in demethylation was observed in psoriatic skin lesions, resulting in the expression of SHP1 isoform II. Psoriasis is a T-cell mediated disease that involves the dysregulation of MAPK and JAK/STAT signaling [81]. Another study has shown that SHP1 deficiency in mice resulted in inflammatory skin disease due to abnormal toll-like receptor (TLR) activation leading to increased IL-1β production in neutrophils [82].

Along with STAT3, SHP1 has been reported to regulate STAT5 and STAT6. STAT5 plays a role in many hematopoietic malignancies. For example, SHP1 mRNA and protein levels were found to be significantly reduced in patients with chronic myelogenous leukemia as a result of hypermethylation of the *PTPN6* promoter, and an in vitro study revealed that the overexpression of SHP1 negatively regulated several signaling pathways, including JAK2/STAT5 signaling [83]. Another recent study showed that phospholipase C β3 (PLC-β3) recruits SHP1 and STAT5 to its C-terminal domain to facilitate the dephosphorylation of STAT5, which inhibits cell proliferation, survival, and differentiation, leading to the suppression of myeloproliferative disease, lymphoma, and other tumors in PLC-β3 knockout mice [84]. Interestingly, PLC-β3 knockout mice can spontaneously develop atopic dermatitis-like skin lesions and severe allergen-induced dermatitis, and these symptoms required the presence of mast cells [85]. These mast cells were sensitive to IL-3 stimulation due to the increased STAT5 activation, which could be reversed by SHP1. STAT6 is involved in IL-4-mediated cellular processes, and SHP1 has been shown to regulate IL-4 induction of STAT6 activation, which may contribute to T-cell homeostasis [86,87,88].

## 6. Src Homology Region 2 Domain-Containing Phosphatase-2 

SHP2 is a ubiquitously expressed, cytosolic non-receptor PTP, encoded by *PTPN11*. It has an identical structure to SHP1 except that its C-terminal tail contains proline-rich domains instead of a nuclear localization sequence (NLS). So, as with SHP1, it is auto-inhibited by the N-terminal SH-2 domains [63]. SHP2 functions as a major regulator of the cell signaling required for cell growth, transformation, differentiation, spreading, migration, and cytoskeleton organization [89,90,91]. It enhances signal transduction through its interactions with different growth factors, scaffolding adaptors, cytokines, and extracellular matrix receptors that possess a SH-2 domain [89,91]. In particular, SHP2 is required for the activation of the Ras GTPase/extracellular signal-regulated kinase (ERK) signaling cascade [90,91,92]. The exact mechanism of activation remains unclear; however, it is known that ERK activation results in the inactivation of the pro-apoptotic proteins Bim (Bcl-2-like protein 11) and BAD (Bcl-2-associated death promoter), members of the Bcl-2 family of proteins. It also has been implicated in the regulation of the Phosphatidylinositol-4,5-bisphosphate 3-kinase (PI3K)/AKT pathway, which results in the promotion of cell survival through the suppression of caspase 3-mediated apoptosis [93].

Germline gain-of-function mutations of *PTPN11* cause ~50% of the cases of the genetic disease Noonan Syndrome, a disorder that causes abnormal development in different parts of the body and is associated with the increased risk of malignancy and leukemia [90,91,92,94]. The rare genetic disease LEOPARD syndrome, a variant of Noonan Syndrome, given the similarities in characteristic symptoms, is also caused by germline mutations in *PTPN11*; however, these missense mutations inactivate SHP2 [94]. SHP2 has been established as a major oncogenic protein, and in fact, *PTPN11* was the first proto-oncogene identified to encode a tyrosine phosphatase [95]. PTPN11 mutations have been identified in lung, breast, and colon cancer, leukemia, neuroblastoma, and melanoma. The dysregulation of SHP2 in cancer cells has been implicated to play a role in tumor invasion and metastasis, apoptosis, cell proliferation, DNA damage repair, and chemoresistance [90,92,96].

Given that SHP2 is primarily known to promote cancer, it seems counter-intuitive that it would function to dephosphorylate STAT3; however, some studies have revealed that SHP2 shows some tumor suppressive capabilities through its inactivation of STAT3. Bard-Chapeau et al. [97] demonstrated that the knockout of SHP2 in hepatocytes of mice caused liver inflammation and necrosis, leading to nodular regenerative hyperplasia. SHP2-deficient mice also presented an increase in spontaneous hepatocellular adenomas and chemically-induced HCCs as a result of an increase in STAT3-mediated inflammatory signaling. In agreement with these findings, SHP2 is downregulated in a small population of human HCCs [97]. Another study showed that SHP2 expression is also repressed in human esophageal squamous cell cancer (ESCC), and SHP2 knockdown results in increased ESCC cell proliferation in vitro and in vivo, which corresponded with a significant increase in phosphorylated STAT3 [98]. Moreover, Huang et al. [99] demonstrated that SHP2 inhibition of colorectal cancer cell proliferation and migration corresponded with the negative regulation of STAT3 by SHP2; more importantly, low expression levels of SHP2 and high expression levels of phosphorylated STAT3 were associated with poor patient prognosis and vice versa. As mentioned above, SHP2 has been shown to cooperate with SHP1 and TC-PTP to dephosphorylate STAT3 in response to UV irradiation as part of an initial protective response against skin carcinogenesis [80]. Additionally, in melanoma cells, the loss of glucose-phosphate dehydrogenase, a key metabolic enzyme that is highly expressed in different cancers, and NADPH oxidase 4 inhibit cell proliferation via the suppression of SHP2 and Src, which regulate STAT3 activation and DNA binding activity, respectively [100].

SHP2 has also been implicated in the regulation of STAT1 and STAT5. STAT1 mediates various biological functions in normal cells, such as cell death, cell growth inhibition, immune system stimulation, and cell differentiation regulation [101]. The knockout of SHP2 in mouse fibroblast cells resulted in enhanced and prolonged STAT1 phosphorylation at both Y701 and S727, induced by interferon (IFN) γ, and an immunoprecipitation assay confirmed that SHP2 interacts directly with STAT1 to negatively regulate it [102]. Interestingly, STAT1 is primarily considered to be a tumor suppressor. Consistent with this hypothesis, Liu et al. found that SHP2 expression is high in human prostate cancer cell lines, which coincided with low STAT1 phosphorylation and decreased T-cell activation [103]. Additionally, SHP2 expression is upregulated in human head and neck cancer tissue, and an in vitro study showed that the loss of SHP2 promoted STAT1 activation, which reduced human leukocyte antigen class I levels, leading to secretion of inflammatory, T-cell-attracting chemokines [104]. With regards to STAT5, Chen et al. demonstrated that SHP2—and not SHP1—directly accelerates the dephosphorylation of STAT5a [105]. It was also demonstrated that a SHP2 deficiency in human CD34^+^ hematopoietic progenitor cells significantly inhibited growth factor-mediated cell survival, proliferation, and differentiation via the downregulation of ERK1/2, AKT, and JAK/STAT5 signaling [83].

## 7. MEG2/Protein Tyrosine Phosphatase Non-Receptor Type 9

PTP non-receptor type 9, otherwise known as PTP-MEG2 or simply, MEG2, is a cytosolic non-transmembrane PTP encoded by *PTPN9* that is comprised of a PTP domain and a unique lipid-binding domain near the N-terminus. This domain has sequence similarity with yeast phosphatidylinositol transfer protein (Sec14p) and cellular retinaldehyde binding protein, and it targets MEG2 to secretory vesicles through its ability to bind to phosphatidylinositol 3,4,5-triphosphate (PIP3) and phosphatidylserine [106,107]. MEG2 is widely expressed in different tissues, such as brain, leukocytes, and endocrine cells [108,109]. However, the functions of PTP-MEG2 have not been completely characterized yet. Some research has demonstrated that it regulates the growth and expansion of erythroid cells, and it regulates embryonic development [110,111]. Other studies have provided useful progress towards identifying a few substrates of MEG2. MEG2 has been shown to dephosphorylate the nerve growth factor (NGF) receptor TrkA at multiple sites, which resulted in the inhibition of TrkA signaling and NGF-mediated cell differentiation [107]. Additionally, MEG2 has been shown to dephosphorylate and deactivate the insulin receptor in hepatocytes, which downregulates insulin signaling [112]. The use of a substrate-trapping mutant revealed that MEG2 dephosphorylates vascular endothelial growth factor (VEGF) receptor 2, which corresponded with decreased VEGF receptor 2 signaling, as evidenced by the reduced expression levels of the downstream molecules IL-6 and IL-8 [106]. Su et al. [113] were the first to show that STAT3 is yet another substrate of MEG2. Co-immunoprecipitation and glutathione S-transferase (GST) pull-down assays demonstrated that MEG2 directly interacts with STAT3 in vitro, and this interaction was confirmed in in vivo mouse brain tissue and human breast cancer cells. Furthermore, MEG2 dephosphorylated STAT3 at Y705 in a time- and dose-dependent manner, and the inactivation of STAT3 resulted in decreased breast tumor growth [113]. Jin et al. further revealed that the STAT3 inhibitor methyllucidone, a natural compound derived from the fruits of *Lindera erythrocarpa* Makino, inhibited STAT3 activation by upregulating MEG2 expression and, thereby, was effective at inhibiting prostate cancer cell survival and proliferation [114].

## 8. T-Cell Protein Tyrosine Phosphatase

TC-PTP (encoded by *PTPN2*) is a non-transmembrane protein belonging to the non-receptor, tyrosine-specific subfamily of PTPs, which also includes PTP1B. TC-PTP is comprised of a conserved catalytic domain that shares a high degree (>72%) of sequence and structural homology with the catalytic domain of PTP-1B and a non-catalytic C-terminal domain. TC-PTP has two splice variants that express two isoenzymes: TC45 (45 kDa) possesses a shorter hydrophilic C-terminal domain containing a NLS and TC48 (48 kDa), which has a hydrophobic endoplasmic reticulum targeting sequence within its C-terminal domain [115,116]. TC45 is the major form of TC-PTP in most species; it is primarily found in the nucleus of most cell types, and shuttles between the nucleus and cytoplasm in response to growth factor and cytokine receptor signaling to dephosphorylate distinct substrates [116,117,118]. Although it is most highly expressed in hematopoietic cells, TC-PTP is ubiquitously expressed in most embryonic and adult tissues [119]. Similar to other PTPs, it regulates major cell processes, such as cell growth and differentiation. For example, insulin, the hormone that maintains glucose homeostasis, triggers the accumulation of TC-PTP in the cytoplasm where it can dephosphorylate the insulin receptor, resulting in the downregulation of insulin signaling [120]. Moreover, TC-PTP has been shown to play unique roles in the immune system [121]. TC-PTP-deficient (*Ptpn2*^−/−^) mice develop thymus atrophy and succumb to anemia and progressive systemic inflammatory disease within the first 2 weeks of birth [122]. Additionally, an increase in pro-inflammatory cytokines, such as IFN-γ, IFN-α, or TNF-α, could be found in the 3 days after birth [123]. Older T-cell-specific TC-PTP-deficient (*Ptpn2*^−/−^) mice suffer from spontaneous autoimmunity corresponding with enhanced activation of both CD4 and CD8 T cells in vivo*,* leading to a reduction in the T-cell receptor (TCR) threshold of activation. In addition, a recent report demonstrated that TC-PTP is a negative regulator of interleukin-7 receptor (IL-7R)-STAT signaling in T-cell progenitors and works as a critical safeguard for efficient T-cell differentiation [124].

**Figure 2:**
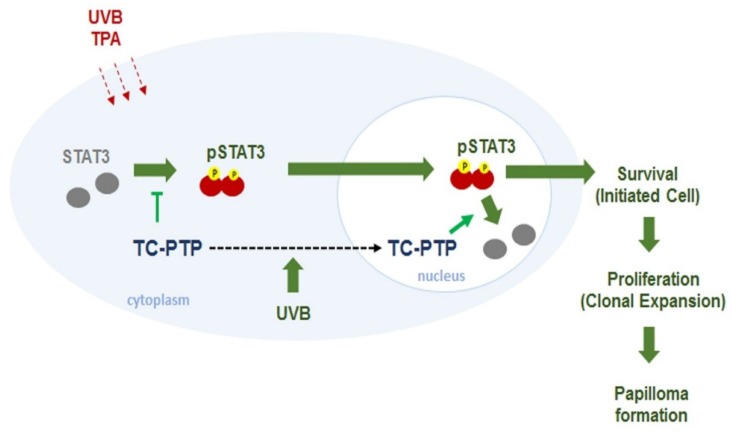
Regulation of STAT3 by T-cell protein tyrosine phosphatase (TC-PTP) in keratinocytes. In response to ultraviolet B (UVB) radiation or 12-*O-*tetradecanoylphorbol-13-acetate (TPA) treatment, TC-PTP dephosphorylates active p(Y)-STAT3 in both the nucleus and cytoplasm of keratinocytes. UVB- or TPA-induced nuclear translocation of TC-PTP by the AKT/14-3-3σ axis can enhance STAT3 dephosphorylation, which can contribute to increased apoptosis and decreased cell proliferation in keratinocytes. This initial cellular response by TC-PTP protects against the development of 7,12-dimethylbenz[a]anthracene (DMBA/TPA)- or UVB-induced skin cancer.

TC-PTP has several substrates such as EGF receptor, JAK, and STAT [125,126]. As previously mentioned, TC-PTP is able to play a regulatory role in insulin signaling. TC-PTP-deficient mice (*Ptpn2*^+/−^) displayed decreased gluconeogenesis as a result of the upregulation of STAT3 signaling and insulin-stimulated AKT signaling in the liver [127]. Also, it has been revealed that TC-PTP has some tumor suppressor capabilities. TC-PTP is deleted in a small proportion of human T-cell acute lymphoblastic leukemia, leading to increased JAK/STAT signaling [128,129]. Another study reported a loss of TC-PTP in triple-negative primary breast cancer, and TC-PTP deficiency in human breast cancer cell lines resulted in increased cell proliferation in vitro and xenograft in vivo via reduced SFK (Src family protein tyrosine kinases) and STAT3 signaling [130]. Studies have shown that STAT3 plays a critical role in the development of either chemically or UVB-induced skin cancer by promoting the survival and proliferation of keratinocytes during carcinogenesis [131,132,133,134,135,136,137]. Our research group has reported extensively on the role of TC-PTP in the regulation of STAT3 signaling in skin carcinogenesis. Our studies have demonstrated that UVB radiation induces TC-PTP nuclear translocation in mouse keratinocytes, which resulted in a significant decrease in cell proliferation corresponding with a decrease in STAT3 phosphorylation [138,139], suggesting that TC-PTP may serve as a tumor suppressor against UVB-mediated skin carcinogenesis. As previously mentioned, TC-PTP is primarily found in the cell nucleus due to a NLS in the C-terminus. However, we revealed that UVB irradiation triggers phosphorylation at the T179 residue of TC-PTP by AKT, which is required for the nuclear translocation of TC-PTP via 14-3-3σ, a protein critical to signal transduction and cell cycle regulation [139,140]. TC-PTP was also implicated in chemically induced skin carcinogenesis. TC-PTP knockout in mouse epidermis (*K14Cre.Ptpn2^fl^*^/*fl*^) led to reduced susceptibility to tumor initiator 7,12-dimethylbenz[a]anthracene (DMBA)-induced apoptosis. Furthermore, TC-PTP deficiency significantly increased epidermal thickness and hyperproliferation following treatment with 12-*O-*tetradecanoylphorbol-13-acetate (TPA), a tumor promoter. During two-stage skin carcinogenesis, the loss of TC-PTP promoted TPA-induced skin carcinogenesis via the upregulation of STAT3 and AKT signaling, whereas the inhibition of STAT3 or AKT recovered the effects of TC-PTP as a tumor suppressor in TC-PTP deficient cell lines (Figure 2) [141]. Our findings implied that TC-PTP is a potential novel target for the prevention of skin cancer through its role in the regulation of STAT3 and AKT signaling.

TC-PTP is the only PTP known to regulate STAT1 other than SHP2. The interaction between STAT1 and TC-PTP may involve associated proteins, such as β-arrestin 1. For instance, during IFN-γ signaling, β-arrestin 1, acting as a scaffold, directly interacts with STAT1 and promotes STAT1 tyrosine dephosphorylation by recruiting TC45 [142]. However, more recent evidence has suggested that β-arrestin 1 does not inhibit STAT1 transcriptional activity nor does it prevent the activation of IFN-γ target genes. Thus, β-arrestin 1 has not been confirmed as a STAT1-interacting protein, and in fact, it has been shown to be negatively regulated in STAT1 signaling [143]. Another interesting study has reported that nuclear TC45 is a major PTP regulator of nuclear STAT1 [144]. It was demonstrated that STAT1 was hyperphosphorylated and activated in TC-PTP-deficient cells. The dephosphorylation of pSTAT1 in TC-PTP deficient mouse fibroblasts was strongly delayed, resulting in the expression of STAT1 target genes. Bussieres-Marmen et al. demonstrated that the knockout of TC-PTP in the intestinal epithelial cells in mice resulted in severe colitis corresponding with increased inflammation and increased cell proliferation via the activation of STAT1 [145]. Interestingly, genetic variations in *PTPN2* have been associated with the chronic inflammatory bowel disease known as Crohn’s disease [146].

## 9. Conclusions

**Figure 3:**
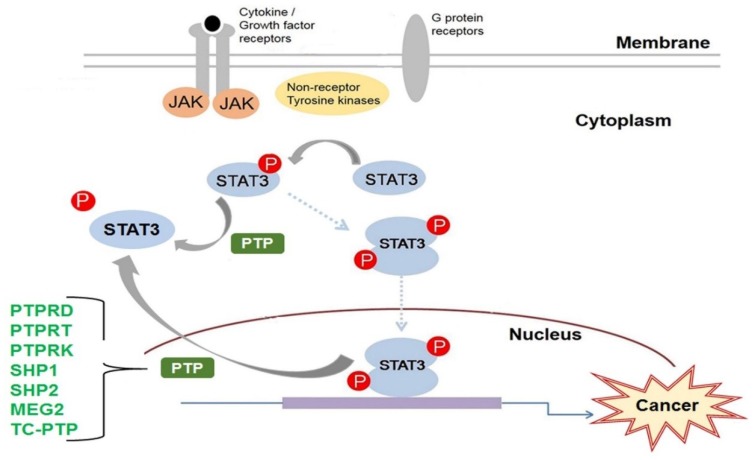
STAT3 dephosphorylation by PTPs in cancer. STAT3 phosphorylation can be initiated by different signaling molecules: cytokine receptors, growth factor receptors, non-receptor tyrosine kinases, or G-protein receptors. Following activation, p(Y)-STAT3 dimerizes and translocates to the nucleus, where it can activate the genes necessary to promote carcinogenesis. The PTPs that have been implicated in the negative regulation of STAT3 in cancer are: PTP receptor-type D (PTPRD), PTP receptor-type T (PTPRT), PTP-receptor-type K (PTPRK), Src homology region 2 (SH-2) domain-containing phosphatase 1 (SHP1), SH-2 domain-containing phosphatase 2 (SHP2), MEG/PTP non-receptor type 9 (PTPN9), and TC-PTP. Dephosphorylation of STAT3 by PTPs can occur in either the cytoplasm or the nucleus. JAK: Janus-activated kinases.

The STAT family of transcription factors are vital to the proper functioning of most cell types. They transduce the signals required for the activation of several key cellular processes. A STAT is activated primarily by tyrosine phosphorylation of its C-terminal tail. Tyrosine phosphorylation is a reversible post-translational modification that regulates proteins in multiple ways; however, during carcinogenesis, the rate and duration of tyrosine phosphorylation can become disrupted by genetic mutation or by the inactivation of important phosphotyrosine regulators. STAT3 is constitutively activated in many cancers; therefore, the mechanisms that regulate STAT3 have been, and continue to be, heavily investigated in order to identify newer and more effective targets for anti-cancer therapies. PTPs are the primary enzymes to dephosphorylate phosphotyrosine proteins. The current research has greatly increased our understanding of how STAT3 is regulated by PTP. To date, seven PTPs have been implicated in the regulation of STAT3: PTPRD, PTPRT, PTPRK, SHP1, SHP2, MEG2, and TC-PTP (Figure 3). However, there are 107 members of the PTP family, many with unknown functions. More research is still needed to unravel the many mechanisms by which PTP regulation may contribute to human disease in order to improve upon our current clinical therapies.

## References

[1] Bowman T., Garcia R., Turkson J., Jove R. (2000). STATs in oncogenesis. Oncogene.

[2] Bromberg J. (2002). STAT proteins and oncogenesis. J. Clin. Investig..

[3] Levy D.E., Lee C.K. (2002). What does STAT3 do?. J. Clin. Investig..

[4] Caldenhoven E., van Dijk T.B., Solari R., Armstrong J., Raaijmakers J.A., Lammers J.W., Koenderman L., de Groot R.P. (1996). STAT3β, a splice variant of transcription factor STAT3, is a dominant negative regulator of transcription. J. Biol. Chem..

[5] Maritano D., Sugrue M.L., Tininini S., Dewilde S., Strobl B., Fu X., Murray-Tait V., Chiarle R., Poli V. (2004). The STAT3 isoforms α and β have unique and specific functions. Nat. Immunol..

[6] Levy D.E., Darnell J.E. (2002). STATs: Transcriptional control and biological impact. Nat. Rev. Mol. Cell. Biol..

[7] Zhang T., Kee W.H., Seow K.T., Fung W., Cao X. (2000). The coiled-coil domain of STAT3 is essential for its SH2 domain-mediated receptor binding and subsequent activation induced by epidermal growth factor and interleukin-6. Mol. Cell. Biol..

[8] Kisseleva T., Bhattacharya S., Braunstein J., Schindler C.W. (2002). Signaling through the JAK/STAT pathway, recent advances and future challenges. Gene.

[9] Seidel H.M., Milocco L.H., Lamb P., Darnell J.E., Stein R.B., Rosen J. (1995). Spacing of palindromic half sites as a determinant of selective STAT (signal transducers and activators of transcription) DNA binding and transcriptional activity. Proc. Natl. Acad. Sci. USA.

[10] Tkach M., Rosemblit C., Rivas M.A., Proietti C.J., Diaz Flaque M.C., Mercogliano M.F., Beguelin W., Maronna E., Guzman P., Gercovich F.G. (2013). p42/p44 MAPK-mediated STAT3Ser727 phosphorylation is required for progestin-induced full activation of STAT3 and breast cancer growth. Endocr. Relat. Cancer.

[11] Lim C.P., Cao X. (2006). Structure, function, and regulation of STAT proteins. Mol. Biosyst..

[12] Wen Z., Zhong Z., Darnell J.E. (1995). Maximal activation of transcription by STAT1 and STAT3 requires both tyrosine and serine phosphorylation. Cell.

[13] Decker T., Kovarik P. (2000). Serine phosphorylation of STATs. Oncogene.

[14] Tammineni P., Anugula C., Mohammed F., Anjaneyulu M., Larner A.C., Sepuri N.B. (2013). The import of the transcription factor STAT3 into mitochondria depends on GRIM-19, a component of the electron transport chain. J. Biol. Chem..

[15] Luo X., Ribeiro M., Bray E.R., Lee D.H., Yungher B.J., Mehta S.T., Thakor K.A., Diaz F., Lee J.K., Moraes C.T. (2016). Enhanced Transcriptional Activity and Mitochondrial Localization of STAT3 Co-induce Axon Regrowth in the Adult Central Nervous System. Cell Rep..

[16] Gough D.J., Corlett A., Schlessinger K., Wegrzyn J., Larner A.C., Levy D.E. (2009). Mitochondrial STAT3 supports Ras-dependent oncogenic transformation. Science.

[17] Wegrzyn J., Potla R., Chwae Y.J., Sepuri N.B., Zhang Q., Koeck T., Derecka M., Szczepanek K., Szelag M., Gornicka A. (2009). Function of mitochondrial STAT3 in cellular respiration. Science.

[18] Lim W.A., Pawson T. (2010). Phosphotyrosine signaling: Evolving a new cellular communication system. Cell.

[19] Hunter T. (2009). Tyrosine phosphorylation: Thirty years and counting. Curr. Opin. Cell Biol..

[20] Julien S.G., Dube N., Hardy S., Tremblay M.L. (2011). Inside the human cancer tyrosine phosphatome. Nat. Rev. Cancer.

[21] Tonks N.K. (1996). Protein tyrosine phosphatases and the control of cellular signaling responses. Adv. Pharmacol..

[22] Wallace M.J., Fladd C., Batt J., Rotin D. (1998). The second catalytic domain of protein tyrosine phosphatase δ (PTP δ) binds to and inhibits the first catalytic domain of PTP σ. Mol. Cell. Biol..

[23] Pulido R., Serra-Pages C., Tang M., Streuli M. (1995). The LAR/PTP δ/PTP σ subfamily of transmembrane protein-tyrosine-phosphatases: Multiple human LAR, PTP δ, and PTP σ isoforms are expressed in a tissue-specific manner and associate with the LAR-interacting protein LIP.1. Proc. Natl. Acad. Sci. USA.

[24] Funato K., Yamazumi Y., Oda T., Akiyama T. (2011). Tyrosine phosphatase PTPRD suppresses colon cancer cell migration in coordination with CD44. Exp. Ther. Med..

[25] Gonzalez-Brito M.R., Bixby J.L. (2006). Differential activities in adhesion and neurite growth of fibronectin type III repeats in the PTP-δ extracellular domain. Int. J. Dev. Neurosci..

[26] Veeriah S., Brennan C., Meng S., Singh B., Fagin J.A., Solit D.B., Paty P.B., Rohle D., Vivanco I., Chmielecki J. (2009). The tyrosine phosphatase PTPRD is a tumor suppressor that is frequently inactivated and mutated in glioblastoma and other human cancers. Proc. Natl. Acad. Sci. USA.

[27] Stallings R.L., Nair P., Maris J.M., Catchpoole D., McDermott M., O’Meara A., Breatnach F. (2006). High-resolution analysis of chromosomal breakpoints and genomic instability identifies PTPRD as a candidate tumor suppressor gene in neuroblastoma. Cancer Res..

[28] Purdie K.J., Lambert S.R., Teh M.T., Chaplin T., Molloy G., Raghavan M., Kelsell D.P., Leigh I.M., Harwood C.A., Proby C.M. (2007). Allelic imbalances and microdeletions affecting the PTPRD gene in cutaneous squamous cell carcinomas detected using single nucleotide polymorphism microarray analysis. Genes Chromosomes Cancer.

[29] Solomon D.A., Kim J.S., Cronin J.C., Sibenaller Z., Ryken T., Rosenberg S.A., Ressom H., Jean W., Bigner D., Yan H. (2008). Mutational inactivation of PTPRD in glioblastoma multiforme and malignant melanoma. Cancer Res..

[30] Ortiz B., Fabius A.W., Wu W.H., Pedraza A., Brennan C.W., Schultz N., Pitter K.L., Bromberg J.F., Huse J.T., Holland E.C. (2014). Loss of the tyrosine phosphatase PTPRD leads to aberrant STAT3 activation and promotes gliomagenesis. Proc. Natl. Acad. Sci. USA.

[31] Peyser N.D., Du Y., Li H., Lui V., Xiao X., Chan T.A., Grandis J.R. (2015). Loss-of-Function PTPRD Mutations Lead to Increased STAT3 Activation and Sensitivity to STAT3 Inhibition in Head and Neck Cancer. PLoS ONE.

[32] Walia V., Prickett T.D., Kim J.S., Gartner J.J., Lin J.C., Zhou M., Rosenberg S.A., Elble R.C., Solomon D.A., Waldman T. (2014). Mutational and functional analysis of the tumor-suppressor PTPRD in human melanoma. Hum. Mutat..

[33] Chan T.A., Heguy A. (2009). The protein tyrosine phosphatase receptor D, a broadly inactivated tumor suppressor regulating STAT function. Cell Cycle.

[34] Scott A., Wang Z. (2011). Tumour suppressor function of protein tyrosine phosphatase receptor-T. Biosci. Rep..

[35] Zhang P., Becka S., Craig S.E., Lodowski D.T., Brady-Kalnay S.M., Wang Z. (2009). Cancer-derived mutations in the fibronectin III repeats of PTPRT/PTPρ inhibit cell-cell aggregation. Cell Commun. Adhes..

[36] Becka S., Zhang P., Craig S.E., Lodowski D.T., Wang Z., Brady-Kalnay S.M. (2010). Characterization of the adhesive properties of the type IIb subfamily receptor protein tyrosine phosphatases. Cell Commun. Adhes..

[37] Yu J., Becka S., Zhang P., Zhang X., Brady-Kalnay S.M., Wang Z. (2008). Tumor-derived extracellular mutations of PTPRT/PTPρ are defective in cell adhesion. Mol. Cancer Res..

[38] Besco J.A., Hooft van Huijsduijnen R., Frostholm A., Rotter A. (2006). Intracellular substrates of brain-enriched receptor protein tyrosine phosphatase ρ (RPTPρ/PTPRT). Brain Res..

[39] Lim S.H., Kwon S.K., Lee M.K., Moon J., Jeong D.G., Park E., Kim S.J., Park B.C., Lee S.C., Ryu S.E. (2009). Synapse formation regulated by protein tyrosine phosphatase receptor T through interaction with cell adhesion molecules and Fyn. EMBO J..

[40] Zhao Y., Zhang X., Guda K., Lawrence E., Sun Q., Watanabe T., Iwakura Y., Asano M., Wei L., Yang Z. (2010). Identification and functional characterization of paxillin as a target of protein tyrosine phosphatase receptor T. Proc. Natl. Acad. Sci. USA.

[41] Zhang X., Guo A., Yu J., Possemato A., Chen Y., Zheng W., Polakiewicz R.D., Kinzler K.W., Vogelstein B., Velculescu V.E. (2007). Identification of STAT3 as a substrate of receptor protein tyrosine phosphatase T. Proc. Natl. Acad. Sci. USA.

[42] Wang Z., Shen D., Parsons D.W., Bardelli A., Sager J., Szabo S., Ptak J., Silliman N., Peters B.A., van der Heijden M.S. (2004). Mutational analysis of the tyrosine phosphatome in colorectal cancers. Science.

[43] Lui V.W., Peyser N.D., Ng P.K., Hritz J., Zeng Y., Lu Y., Li H., Wang L., Gilbert B.R., General I.J. (2014). Frequent mutation of receptor protein tyrosine phosphatases provides a mechanism for STAT3 hyperactivation in head and neck cancer. Proc. Natl. Acad. Sci. USA.

[44] Zhao Y., Scott A., Zhang P., Hao Y., Feng X., Somasundaram S., Khalil A.M., Willis J., Wang Z. (2017). Regulation of paxillin-p130-PI3K-AKT signaling axis by Src and PTPRT impacts colon tumorigenesis. Oncotarget.

[45] Peyser N.D., Freilino M., Wang L., Zeng Y., Li H., Johnson D.E., Grandis J.R. (2016). Frequent promoter hypermethylation of PTPRT increases STAT3 activation and sensitivity to STAT3 inhibition in head and neck cancer. Oncogene.

[46] Qi J., Li N., Fan K., Yin P., Zhao C., Li Z., Lin Y., Wang L., Zha X. (2014). β1,6 GlcNAc branches-modified PTPRT attenuates its activity and promotes cell migration by STAT3 pathway. PLoS ONE.

[47] Sap J., Jiang Y.P., Friedlander D., Grumet M., Schlessinger J. (1994). Receptor tyrosine phosphatase R-PTP-κ mediates homophilic binding. Mol. Cell. Biol..

[48] Wang S.E., Wu F.Y., Shin I., Qu S., Arteaga C.L. (2005). Transforming growth factor β (TGF-β)-Smad target gene protein tyrosine phosphatase receptor type κ is required for TGF-β function. Mol. Cell. Biol..

[49] Drosopoulos N.E., Walsh F.S., Doherty P. (1999). A soluble version of the receptor-like protein tyrosine phosphatase κ stimulates neurite outgrowth via a Grb2/MEK1-dependent signaling cascade. Mol. Cell. Neurosci..

[50] Kose H., Sakai T., Tsukumo S., Wei K., Yamada T., Yasutomo K., Matsumoto K. (2007). Maturational arrest of thymocyte development is caused by a deletion in the receptor-like protein tyrosine phosphatase κ gene in LEC rats. Genomics.

[51] Schnekenburger J., Mayerle J., Kruger B., Buchwalow I., Weiss F.U., Albrecht E., Samoilova V.E., Domschke W., Lerch M.M. (2005). Protein tyrosine phosphatase κ and SHP-1 are involved in the regulation of cell-cell contacts at adherens junctions in the exocrine pancreas. Gut.

[52] Fuchs M., Muller T., Lerch M.M., Ullrich A. (1996). Association of human protein-tyrosine phosphatase κ with members of the armadillo family. J. Biol. Chem..

[53] Sun P.H., Ye L., Mason M.D., Jiang W.G. (2013). Protein tyrosine phosphatase κ (PTPRK) is a negative regulator of adhesion and invasion of breast cancer cells, and associates with poor prognosis of breast cancer. J. Cancer Res. Clin. Oncol..

[54] Nakamura M., Kishi M., Sakaki T., Hashimoto H., Nakase H., Shimada K., Ishida E., Konishi N. (2003). Novel tumor suppressor loci on 6q22-23 in primary central nervous system lymphomas. Cancer Res..

[55] Starr T.K., Allaei R., Silverstein K.A., Staggs R.A., Sarver A.L., Bergemann T.L., Gupta M., O’Sullivan M.G., Matise I., Dupuy A.J. (2009). A transposon-based genetic screen in mice identifies genes altered in colorectal cancer. Science.

[56] Sun P.H., Ye L., Mason M.D., Jiang W.G. (2013). Receptor-like protein tyrosine phosphatase κ negatively regulates the apoptosis of prostate cancer cells via the JNK pathway. Int. J. Oncol..

[57] Agarwal S., Al-Keilani M.S., Alqudah M.A., Sibenaller Z.A., Ryken T.C., Assem M. (2013). Tumor derived mutations of protein tyrosine phosphatase receptor type K affect its function and alter sensitivity to chemotherapeutics in glioma. PLoS ONE.

[58] Xu Y., Tan L.J., Grachtchouk V., Voorhees J.J., Fisher G.J. (2005). Receptor-type protein-tyrosine phosphatase-κ regulates epidermal growth factor receptor function. J. Biol. Chem..

[59] Wang C., Yang Y., Yang Z., Liu M., Li Z., Sun L., Mei C., Chen H., Chen L., Wang L. (2009). EGF-mediated migration signaling activated by N-acetylglucosaminyltransferase-V via receptor protein tyrosine phosphatase κ. Arch. Biochem. Biophys..

[60] Xu Y., Xue S., Zhou J., Voorhees J.J., Fisher G.J. (2015). Notch and TGF-β pathways cooperatively regulate receptor protein tyrosine phosphatase-κ (PTPRK) gene expression in human primary keratinocytes. Mol. Biol. Cell.

[61] Chen Y.W., Guo T., Shen L., Wong K.Y., Tao Q., Choi W.W., Au-Yeung R.K., Chan Y.P., Wong M.L., Tang J.C. (2015). Receptor-type tyrosine-protein phosphatase κ directly targets STAT3 activation for tumor suppression in nasal NK/T-cell lymphoma. Blood.

[62] Zhang J., Somani A.K., Siminovitch K.A. (2000). Roles of the SHP-1 tyrosine phosphatase in the negative regulation of cell signalling. Semin. Immunol..

[63] Neel B.G., Gu H., Pao L. (2003). The ‘Shp’ing news: SH2 domain-containing tyrosine phosphatases in cell signaling. Trends Biochem. Sci..

[64] Wang W., Liu L., Song X., Mo Y., Komma C., Bellamy H.D., Zhao Z.J., Zhou G.W. (2011). Crystal structure of human protein tyrosine phosphatase SHP-1 in the open conformation. J. Cell. Biochem..

[65] Wu C., Sun M., Liu L., Zhou G.W. (2003). The function of the protein tyrosine phosphatase SHP-1 in cancer. Gene.

[66] Yang J., Liu L., He D., Song X., Liang X., Zhao Z.J., Zhou G.W. (2003). Crystal structure of human protein-tyrosine phosphatase SHP-1. J. Biol. Chem..

[67] Frank C., Burkhardt C., Imhof D., Ringel J., Zschornig O., Wieligmann K., Zacharias M., Bohmer F.D. (2004). Effective dephosphorylation of Src substrates by SHP-1. J. Biol. Chem..

[68] Han Y., Amin H.M., Franko B., Frantz C., Shi X., Lai R. (2006). Loss of SHP1 enhances JAK3/STAT3 signaling and decreases proteosome degradation of JAK3 and NPM-ALK in ALK^+^ anaplastic large-cell lymphoma. Blood.

[69] Khoury J.D., Rassidakis G.Z., Medeiros L.J., Amin H.M., Lai R. (2004). Methylation of *SHP1* gene and loss of SHP1 protein expression are frequent in systemic anaplastic large cell lymphoma. Blood.

[70] Chim C.S., Fung T.K., Cheung W.C., Liang R., Kwong Y.L. (2004). SOCS1 and SHP1 hypermethylation in multiple myeloma: Implications for epigenetic activation of the JAK/STAT pathway. Blood.

[71] Oka T., Ouchida M., Koyama M., Ogama Y., Takada S., Nakatani Y., Tanaka T., Yoshino T., Hayashi K., Ohara N. (2002). Gene silencing of the tyrosine phosphatase SHP1 gene by aberrant methylation in leukemias/lymphomas. Cancer Res..

[72] Koyama M., Oka T., Ouchida M., Nakatani Y., Nishiuchi R., Yoshino T., Hayashi K., Akagi T., Seino Y. (2003). Activated proliferation of B-cell lymphomas/leukemias with the *SHP1* gene silencing by aberrant CpG methylation. Lab. Investig..

[73] Witkiewicz A., Raghunath P., Wasik A., Junkins-Hopkins J.M., Jones D., Zhang Q., Odum N., Wasik M.A. (2007). Loss of SHP-1 tyrosine phosphatase expression correlates with the advanced stages of cutaneous T-cell lymphoma. Hum. Pathol..

[74] Zhang Q., Wang H.Y., Marzec M., Raghunath P.N., Nagasawa T., Wasik M.A. (2005). STAT3- and DNA methyltransferase 1-mediated epigenetic silencing of SHP-1 tyrosine phosphatase tumor suppressor gene in malignant T lymphocytes. Proc. Natl. Acad. Sci. USA.

[75] Demosthenous C., Han J.J., Hu G., Stenson M., Gupta M. (2015). Loss of function mutations in PTPN6 promote STAT3 deregulation via JAK3 kinase in diffuse large B-cell lymphoma. Oncotarget.

[76] Ahn K.S., Sethi G., Sung B., Goel A., Ralhan R., Aggarwal B.B. (2008). Guggulsterone, a farnesoid X receptor antagonist, inhibits constitutive and inducible STAT3 activation through induction of a protein tyrosine phosphatase SHP-1. Cancer Res..

[77] Tai W.T., Cheng A.L., Shiau C.W., Liu C.Y., Ko C.H., Lin M.W., Chen P.J., Chen K.F. (2012). Dovitinib induces apoptosis and overcomes sorafenib resistance in hepatocellular carcinoma through SHP-1-mediated inhibition of STAT3. Mol. Cancer Ther..

[78] Chen K.F., Tai W.T., Hsu C.Y., Huang J.W., Liu C.Y., Chen P.J., Kim I., Shiau C.W. (2012). Blockade of STAT3 activation by sorafenib derivatives through enhancing SHP-1 phosphatase activity. Eur. J. Med. Chem..

[79] Sandur S.K., Pandey M.K., Sung B., Aggarwal B.B. (2010). 5-hydroxy-2-methyl-1,4-naphthoquinone, a vitamin K3 analogue, suppresses STAT3 activation pathway through induction of protein tyrosine phosphatase, SHP-1: Potential role in chemosensitization. Mol. Cancer Res..

[80] Kim D.J., Tremblay M.L., Digiovanni J. (2010). Protein tyrosine phosphatases, TC-PTP, SHP1, and SHP2, cooperate in rapid dephosphorylation of STAT3 in keratinocytes following UVB irradiation. PLoS ONE.

[81] Ruchusatsawat K., Wongpiyabovorn J., Shuangshoti S., Hirankarn N., Mutirangura A. (2006). SHP-1 promoter 2 methylation in normal epithelial tissues and demethylation in psoriasis. J. Mol. Med. (Berl.).

[82] Croker B.A., Lewis R.S., Babon J.J., Mintern J.D., Jenne D.E., Metcalf D., Zhang J.G., Cengia L.H., O’Donnell J.A., Roberts A.W. (2011). Neutrophils require SHP1 to regulate IL-1β production and prevent inflammatory skin disease. J. Immunol..

[83] Li L., Modi H., McDonald T., Rossi J., Yee J.K., Bhatia R. (2011). A critical role for SHP2 in STAT5 activation and growth factor-mediated proliferation, survival, and differentiation of human CD34^+^ cells. Blood.

[84] Xiao W., Hong H., Kawakami Y., Kato Y., Wu D., Yasudo H., Kimura A., Kubagawa H., Bertoli L.F., Davis R.S. (2009). Tumor suppression by phospholipase C-β3 via SHP-1-mediated dephosphorylation of STAT5. Cancer Cell.

[85] Ando T., Xiao W., Gao P., Namiranian S., Matsumoto K., Tomimori Y., Hong H., Yamashita H., Kimura M., Kashiwakura J. (2014). Critical role for mast cell STAT5 activity in skin inflammation. Cell Rep..

[86] Hanson E.M., Dickensheets H., Qu C.K., Donnelly R.P., Keegan A.D. (2003). Regulation of the dephosphorylation of STAT6. Participation of Tyr-713 in the interleukin-4 receptor α, the tyrosine phosphatase SHP-1, and the proteasome. J. Biol. Chem..

[87] Huang Z., Coleman J.M., Su Y., Mann M., Ryan J., Shultz L.D., Huang H. (2005). SHP-1 regulates STAT6 phosphorylation and IL-4-mediated function in a cell type-specific manner. Cytokine.

[88] Johnson D.J., Pao L.I., Dhanji S., Murakami K., Ohashi P.S., Neel B.G. (2013). Shp1 regulates T cell homeostasis by limiting IL-4 signals. J. Exp. Med..

[89] Yu D.H., Qu C.K., Henegariu O., Lu X., Feng G.S. (1998). Protein-tyrosine phosphatase Shp-2 regulates cell spreading, migration, and focal adhesion. J. Biol. Chem..

[90] Bentires-Alj M., Paez J.G., David F.S., Keilhack H., Halmos B., Naoki K., Maris J.M., Richardson A., Bardelli A., Sugarbaker D.J. (2004). Activating mutations of the noonan syndrome-associated *SHP2*/*PTPN11* gene in human solid tumors and adult acute myelogenous leukemia. Cancer Res..

[91] Mohi M.G., Neel B.G. (2007). The role of SHP2 (PTPN11) in cancer. Curr. Opin. Genet. Dev..

[92] Ostman A., Hellberg C., Bohmer F.D. (2006). Protein-tyrosine phosphatases and cancer. Nat. Rev. Cancer.

[93] Ivins Zito C., Kontaridis M.I., Fornaro M., Feng G.S., Bennett A.M. (2004). SHP-2 regulates the phosphatidylinositide 3’-kinase/Akt pathway and suppresses caspase 3-mediated apoptosis. J. Cell. Physiol..

[94] Kontaridis M.I., Swanson K.D., David F.S., Barford D., Neel B.G. (2006). PTPN11 (SHP2) mutations in LEOPARD syndrome have dominant negative, not activating, effects. J. Biol. Chem..

[95] Chan R.J., Feng G.S. (2007). PTPN11 is the first identified proto-oncogene that encodes a tyrosine phosphatase. Blood.

[96] Zhang J., Zhang F., Niu R. (2015). Functions of SHP2 in cancer. J. Cell. Mol. Med..

[97] Bard-Chapeau E.A., Li S., Ding J., Zhang S.S., Zhu H.H., Princen F., Fang D.D., Han T., Bailly-Maitre B., Poli V. (2011). PTPN11/SHP2 acts as a tumor suppressor in hepatocellular carcinogenesis. Cancer Cell.

[98] Qi C., Han T., Tang H., Huang K., Min J., Li J., Ding X., Xu Z. (2017). SHP2 Inhibits Proliferation of Esophageal Squamous Cell Cancer via Dephosphorylation of STAT3. Int. J. Mol. Sci..

[99] Huang Y., Wang J., Cao F., Jiang H., Li A., Li J., Qiu L., Shen H., Chang W., Zhou C. (2017). SHP2 associates with nuclear localization of STAT3: Significance in progression and prognosis of colorectal cancer. Sci. Rep..

[100] Cai T., Kuang Y., Zhang C., Zhang Z., Chen L., Li B., Li Y., Wang Y., Yang H., Han Q. (2015). Glucose-6-phosphate dehydrogenase and NADPH oxidase 4 control STAT3 activity in melanoma cells through a pathway involving reactive oxygen species, c-SRC and SHP2. Am. J. Cancer Res..

[101] Ramana C.V., Chatterjee-Kishore M., Nguyen H., Stark G.R. (2000). Complex roles of STAT1 in regulating gene expression. Oncogene.

[102] Wu T.R., Hong Y.K., Wang X.D., Ling M.Y., Dragoi A.M., Chung A.S., Campbell A.G., Han Z.Y., Feng G.S., Chin Y.E. (2002). SHP-2 is a dual-specificity phosphatase involved in STAT1 dephosphorylation at both tyrosine and serine residues in nuclei. J. Biol. Chem..

[103] Liu Z., Zhao Y., Fang J., Cui R., Xiao Y., Xu Q. (2017). SHP2 negatively regulates HLA-ABC and PD-L1 expression via STAT1 phosphorylation in prostate cancer cells. Oncotarget.

[104] Leibowitz M.S., Srivastava R.M., Andrade Filho P.A., Egloff A.M., Wang L., Seethala R.R., Ferrone S., Ferris R.L. (2013). SHP2 is overexpressed and inhibits pSTAT1-mediated APM component expression, T-cell attracting chemokine secretion, and CTL recognition in head and neck cancer cells. Clin. Cancer Res..

[105] Chen Y., Wen R., Yang S., Schuman J., Zhang E.E., Yi T., Feng G.S., Wang D. (2003). Identification of SHP-2 as a STAT5A phosphatase. J. Biol. Chem..

[106] Hao Q., Samten B., Ji H.L., Zhao Z.J., Tang H. (2012). Tyrosine phosphatase PTP-MEG2 negatively regulates vascular endothelial growth factor receptor signaling and function in endothelial cells. Am. J. Physiol. Cell Physiol..

[107] Zhang D., Marlin M.C., Liang Z., Ahmad M., Ashpole N.M., Sonntag W.E., Zhao Z.J., Li G. (2016). The Protein Tyrosine Phosphatase MEG2 Regulates the Transport and Signal Transduction of Tropomyosin Receptor Kinase A. J. Biol. Chem..

[108] Gu M., Warshawsky I., Majerus P.W. (1992). Cloning and expression of a cytosolic megakaryocyte protein-tyrosine-phosphatase with sequence homology to retinaldehyde-binding protein and yeast SEC14p. Proc. Natl. Acad. Sci. USA.

[109] Saito K., Williams S., Bulankina A., Honing S., Mustelin T. (2007). Association of protein-tyrosine phosphatase MEG2 via its Sec14p homology domain with vesicle-trafficking proteins. J. Biol. Chem..

[110] Xu M.J., Sui X., Zhao R., Dai C., Krantz S.B., Zhao Z.J. (2003). PTP-MEG2 is activated in polycythemia vera erythroid progenitor cells and is required for growth and expansion of erythroid cells. Blood.

[111] Wang Y., Vachon E., Zhang J., Cherepanov V., Kruger J., Li J., Saito K., Shannon P., Bottini N., Huynh H. (2005). Tyrosine phosphatase MEG2 modulates murine development and platelet and lymphocyte activation through secretory vesicle function. J. Exp. Med..

[112] Cho C.Y., Koo S.H., Wang Y., Callaway S., Hedrick S., Mak P.A., Orth A.P., Peters E.C., Saez E., Montminy M. (2006). Identification of the tyrosine phosphatase PTP-MEG2 as an antagonist of hepatic insulin signaling. Cell Metab..

[113] Su F., Ren F., Rong Y., Wang Y., Geng Y., Wang Y., Feng M., Ju Y., Li Y., Zhao Z.J. (2012). Protein tyrosine phosphatase Meg2 dephosphorylates signal transducer and activator of transcription 3 and suppresses tumor growth in breast cancer. Breast Cancer Res..

[114] Jin Y., Kim Y.H., Park J.Y., Lee Y.J., Oh H.M., Choi S.K., Han D.C., Kwon B.M. (2018). Methyllucidone inhibits STAT3 activity by regulating the expression of the protein tyrosine phosphatase MEG2 in DU145 prostate carcinoma cells. Bioorg Med. Chem. Lett..

[115] Jia Z., Barford D., Flint A.J., Tonks N.K. (1995). Structural basis for phosphotyrosine peptide recognition by protein tyrosine phosphatase 1B. Science.

[116] Bourdeau A., Dube N., Tremblay M.L. (2005). Cytoplasmic protein tyrosine phosphatases, regulation and function: The roles of PTP1B and TC-PTP. Curr. Opin. Cell Biol..

[117] Tillmann U., Wagner J., Boerboom D., Westphal H., Tremblay M.L. (1994). Nuclear localization and cell cycle regulation of a murine protein tyrosine phosphatase. Mol. Cell. Biol..

[118] Kamatkar S., Radha V., Nambirajan S., Reddy R.S., Swarup G. (1996). Two splice variants of a tyrosine phosphatase differ in substrate specificity, DNA binding, and subcellular location. J. Biol. Chem..

[119] Cool D.E., Tonks N.K., Charbonneau H., Walsh K.A., Fischer E.H., Krebs E.G. (1989). cDNA isolated from a human T-cell library encodes a member of the protein-tyrosine-phosphatase family. Proc. Natl. Acad. Sci. USA.

[120] Galic S., Klingler-Hoffmann M., Fodero-Tavoletti M.T., Puryer M.A., Meng T.C., Tonks N.K., Tiganis T. (2003). Regulation of insulin receptor signaling by the protein tyrosine phosphatase TCPTP. Mol. Cell. Biol..

[121] Simoncic P.D., McGlade C.J., Tremblay M.L. (2006). PTP1B and TC-PTP: Novel roles in immune-cell signaling. Can. J. Physiol. Pharmacol..

[122] You-Ten K.E., Muise E.S., Itie A., Michaliszyn E., Wagner J., Jothy S., Lapp W.S., Tremblay M.L. (1997). Impaired bone marrow microenvironment and immune function in T cell protein tyrosine phosphatase-deficient mice. J. Exp. Med..

[123] Heinonen K.M., Nestel F.P., Newell E.W., Charette G., Seemayer T.A., Tremblay M.L., Lapp W.S. (2004). T-cell protein tyrosine phosphatase deletion results in progressive systemic inflammatory disease. Blood.

[124] Pike K.A., Hatzihristidis T., Bussieres-Marmen S., Robert F., Desai N., Miranda-Saavedra D., Pelletier J., Tremblay M.L. (2017). TC-PTP regulates the IL-7 transcriptional response during murine early T cell development. Sci. Rep..

[125] Tiganis T., Kemp B.E., Tonks N.K. (1999). The protein-tyrosine phosphatase TCPTP regulates epidermal growth factor receptor-mediated and phosphatidylinositol 3-kinase-dependent signaling. J. Biol. Chem..

[126] Simoncic P.D., Lee-Loy A., Barber D.L., Tremblay M.L., McGlade C.J. (2002). The T cell protein tyrosine phosphatase is a negative regulator of janus family kinases 1 and 3. Curr. Biol..

[127] Fukushima A., Loh K., Galic S., Fam B., Shields B., Wiede F., Tremblay M.L., Watt M.J., Andrikopoulos S., Tiganis T. (2010). T-cell protein tyrosine phosphatase attenuates STAT3 and insulin signaling in the liver to regulate gluconeogenesis. Diabetes.

[128] Kleppe M., Lahortiga I., El Chaar T., De Keersmaecker K., Mentens N., Graux C., Van Roosbroeck K., Ferrando A.A., Langerak A.W., Meijerink J.P. (2010). Deletion of the protein tyrosine phosphatase gene PTPN2 in T-cell acute lymphoblastic leukemia. Nat. Genet..

[129] Kleppe M., Tousseyn T., Geissinger E., Kalender Atak Z., Aerts S., Rosenwald A., Wlodarska I., Cools J. (2011). Mutation analysis of the tyrosine phosphatase PTPN2 in Hodgkin’s lymphoma and T-cell non-Hodgkin’s lymphoma. Haematologica.

[130] Shields B.J., Wiede F., Gurzov E.N., Wee K., Hauser C., Zhu H.J., Molloy T.J., O’Toole S.A., Daly R.J., Sutherland R.L. (2013). TCPTP regulates SFK and STAT3 signaling and is lost in triple-negative breast cancers. Mol. Cell. Biol..

[131] Chan K.S., Sano S., Kataoka K., Abel E., Carbajal S., Beltran L., Clifford J., Peavey M., Shen J., Digiovanni J. (2008). Forced expression of a constitutively active form of STAT3 in mouse epidermis enhances malignant progression of skin tumors induced by two-stage carcinogenesis. Oncogene.

[132] Chan K.S., Sano S., Kiguchi K., Anders J., Komazawa N., Takeda J., DiGiovanni J. (2004). Disruption of STAT3 reveals a critical role in both the initiation and the promotion stages of epithelial carcinogenesis. J. Clin. Investig..

[133] Kim D.J., Chan K.S., Sano S., Digiovanni J. (2007). Signal transducer and activator of transcription 3 (STAT3) in epithelial carcinogenesis. Mol. Carcinog.

[134] Kim D.J., Kataoka K., Rao D., Kiguchi K., Cotsarelis G., Digiovanni J. (2009). Targeted disruption of stat3 reveals a major role for follicular stem cells in skin tumor initiation. Cancer Res..

[135] Kim D.J., Angel J.M., Sano S., DiGiovanni J. (2009). Constitutive activation and targeted disruption of signal transducer and activator of transcription 3 (STAT3) in mouse epidermis reveal its critical role in UVB-induced skin carcinogenesis. Oncogene.

[136] Kataoka K., Kim D.J., Carbajal S., Clifford J.L., DiGiovanni J. (2008). Stage-specific disruption of STAT3 demonstrates a direct requirement during both the initiation and promotion stages of mouse skin tumorigenesis. Carcinogenesis.

[137] Kim M., Baek M., Kim D.J. (2017). Protein Tyrosine Signaling and its Potential Therapeutic Implications in Carcinogenesis. Curr. Pharm. Des..

[138] Lee H., Morales L.D., Slaga T.J., Kim D.J. (2015). Activation of T-cell protein-tyrosine phosphatase suppresses keratinocyte survival and proliferation following UVB irradiation. J. Biol. Chem..

[139] Kim M., Morales L.D., Baek M., Slaga T.J., DiGiovanni J., Kim D.J. (2017). UVB-induced nuclear translocation of TC-PTP by AKT/14-3-3σ axis inhibits keratinocyte survival and proliferation. Oncotarget.

[140] Kim M., Morales L.D., Kim D.J. (2017). TC-PTP nuclear trafficking in keratinocytes. Aging (Albany NY).

[141] Lee H., Kim M., Baek M., Morales L.D., Jang I.S., Slaga T.J., DiGiovanni J., Kim D.J. (2017). Targeted disruption of TC-PTP in the proliferative compartment augments STAT3 and AKT signaling and skin tumor development. Sci. Rep..

[142] Mo W., Zhang L., Yang G., Zhai J., Hu Z., Chen Y., Chen X., Hui L., Huang R., Hu G. (2008). Nuclear β-arrestin1 functions as a scaffold for the dephosphorylation of STAT1 and moderates the antiviral activity of IFN-γ. Mol. Cell.

[143] Pelzel C., Begitt A., Wenta N., Vinkemeier U. (2013). Evidence against a role for β-arrestin1 in STAT1 dephosphorylation and the inhibition of interferon-γ signaling. Mol. Cell.

[144] ten Hoeve J., de Jesus Ibarra-Sanchez M., Fu Y., Zhu W., Tremblay M., David M., Shuai K. (2002). Identification of a nuclear STAT1 protein tyrosine phosphatase. Mol. Cell. Biol..

[145] Bussieres-Marmen S., Vinette V., Gungabeesoon J., Aubry I., Perez-Quintero L.A., Tremblay M.L. (2018). Loss of T-cell protein tyrosine phosphatase in the intestinal epithelium promotes local inflammation by increasing colonic stem cell proliferation. Cell. Mol. Immunol..

[146] Festen E.A., Goyette P., Green T., Boucher G., Beauchamp C., Trynka G., Dubois P.C., Lagace C., Stokkers P.C., Hommes D.W. (2011). A meta-analysis of genome-wide association scans identifies IL18RAP, PTPN2, TAGAP, and PUS10 as shared risk loci for Crohn’s disease and celiac disease. PLoS Genet..

